# Building capacity for medical device assessment: online training for Ukrainian experts

**DOI:** 10.1017/S026646232610350X

**Published:** 2026-02-16

**Authors:** Olena Filiniuk, Antonio Migliore, Rossella Di Bidino, Nicola Vicari, Rebecca Kohler, Rabia Sucu

**Affiliations:** 1 Management Sciences for Health, Ukraine; 2 Health Technology Assessment International, Canada; 3 Department of Health Technologies and Innovation, Fondazione Policlinico Universitario Agostino Gemelli IRCCS, Italy; 4 Graduate School of Health Economics and Management, Università Cattolica del Sacro Cuore (ALTEMS), Italy; 5 Management Sciences for Health, USA

**Keywords:** health technology assessment, medical devices, Ukraine, capacity building, online training

## Abstract

As Ukraine works to modernize its healthcare system and align with international standards, integrating health technology assessment (HTA) into the national decision-making process has become a strategic priority. While HTA practices for pharmaceuticals have advanced, substantial gaps persist in the evaluation of medical devices (MDs), which require distinct methodological approaches given their characteristics. This article presents the rationale, development, implementation, and results of a comprehensive online training program aimed at building national capacity in the MD assessment. The program targeted 71 Ukrainian professionals with prior experience in pharmaceutical evaluation but limited exposure to MD. Through 40 hours of live online sessions delivered from September to December 2024, the course emphasized interactive learning, contextual adaptation, and international best practices. Assessments showed significant knowledge gains, with 87 percent of participants completing the program successfully. The initiative demonstrates that targeted, competency-based training can enhance national HTA capacity and may serve as a model for other transitioning healthcare systems.

## Background

Health technology assessment (HTA) is a crucial tool for informing evidence-based policy decisions regarding the adoption and reimbursement of health technologies (HTs) (1) and is widely recognized as an essential component of modern healthcare systems.

In Ukraine, HTA has become increasingly integrated into the national health policy framework, specifically to substantiate key policy decisions. This integration was accelerated by the adoption of the reimbursement framework under the Ministry of Health (MOH) (2). However, these efforts have primarily focused on pharmaceuticals, creating a significant gap in the evaluation of MD, especially given the limited resources for conducting HTA (3).

The MD market in Ukraine is experiencing rapid growth, driven by the introduction of numerous new and innovative HT across various healthcare areas and the acceleration of domestic MD development (4). Considering that the MD market is comparable in scale to the pharmaceutical sector in Ukraine, and that a substantial portion of the state budget is allocated to the procurement of MD, amendments were introduced in December 2023 to the Resolution of the Cabinet of Ministers of Ukraine dated December 23, 2020, No. 1300 “*On Approval of the Procedure for Conducting State Health Technology Assessment.*” These amendments stipulate that, by January 1, 2024, methodological guidelines for conducting state HTA of MD must be developed and approved. In line with this directive, Ukraine has been actively working on the development of specific methodological guidance for HTA for MD. A draft guideline has already been prepared, representing a significant step toward institutionalizing HTA for MD, and is currently under review by relevant national interest holders.

At this stage, the application of HTA for MD in Ukraine remains voluntary and is primarily intended to support informed decision-making in public procurement and healthcare planning.

Conducting HTA for MD often differs significantly from pharmaceuticals in terms of evaluation criteria, evidence requirements, and life cycle management. Assessing MD presents unique challenges due to its iterative nature, with frequent post-market design modifications. Clinical evidence at launch is often limited, lacking randomized controlled trials (5). Therefore, real-world data plays a crucial role. Unlike pharmaceuticals, MDs follow different regulatory pathways, relying more on post-market surveillance. Their cost-effectiveness assessment requires tailored approaches, as pricing and adoption patterns differ from those of pharmaceuticals (6). Additionally, factors like learning curves, incremental innovation, dynamic pricing and organizational impact vary by device type (7).

Recognizing these challenges, the Ukrainian government has sought to expand HTA expertise beyond pharmaceuticals, ensuring that decision-makers have the tools to evaluate MD rigorously. Findings indicate that structured capacity-building programs (8) can significantly enhance the ability of local experts to evaluate MD, leading to improved decision-making and alignment with international best practices (2). To address this need, the MOH requested the Management Science of Health (MSH), through the Safe, Affordable, and Effective Medicines for Ukrainians (SAFEMed) project, to organize an online training for Ukrainian HTA experts focused on building capacity for assessing MD. SAFEMed has been working in Ukraine since 2017 in close partnership with the Government of Ukraine to modernize and strengthen the country’s pharmaceutical governance and financing systems. MSH contracted HTA International (HTAi) to undertake this work. This initiative aims to equip participants with the practical tools, methodological knowledge, and international best practices necessary to support evidence-based evaluation and decision-making related to MD in Ukraine.

This article reports on a contract between MSH and HTAi, which involved designing and delivering an online training course for Ukrainian HTA professionals. These experts had experience in pharmaceutical evaluation but lacked formal training in assessing MD. The training initiative was launched against the backdrop of Ukraine’s healthcare reforms and its efforts to integrate HTA more comprehensively into decision-making.

## Approach to the program development

HTAi developed the training program through a structured, multiphase approach that included needs assessment, curriculum design, delivery and evaluation. At the onset, a detailed training needs assessment identified competency gaps and resulted in defined learning objectives. This foundational process involved extensive consultations with the SAFEMed team and international and local experts, including medical doctors and pharmacists with HTA expertise, to ensure the curriculum was contextually relevant to the Ukrainian healthcare environment. Findings indicated that while most participants were familiar with pharmaceutical HTA, few had experience or formal education in assessing MD. Based on these findings, the curriculum was designed to equip participants with the skills to understand how HTA supports decision-making for MD, critically assess its current status and conduct evaluations with a medium level of autonomy (9). It also emphasized adapting assessments to national contexts and keeping up with evolving HTA methodologies. Special attention was given to Ukraine’s local context, where HTA for MD is still developing, by incorporating sessions on adapting international assessments and emergency preparedness (10).

The course was delivered entirely online via Zoom and included simultaneous interpretation, a key feature that ensured language was not a barrier to participation. This allowed for broad geographic participation and flexibility. Each session included live lectures, segments for questions and answers, case studies and home tasks (11). Case studies and home tasks gave participants the opportunity to independently navigate each phase of an assessment. Solutions to the home tasks were provided and discussed collectively. To support learning, participants received supplementary materials, including peer-reviewed articles, HTA reports and analytical tools. The course material was managed through a shared folder to which all participants had access. To ensure that the program was contemporary and had practical applications, the curriculum included modules on digital health, disinvestment and emergency preparedness.

## Faculty and participants

The program brought together a diverse faculty team (11 lecturers from 8 countries), including experts in various HTA domains, all having at least 15 years of individual experience in HTA, previous publications on the lecture’s topic and current or previous leadership roles in HTAi (e.g., Interest Group chair and advisory committee member). Their collective expertise ensured comprehensive coverage of technical content and enriched participant learning through real-world insights and diverse perspectives. The multidisciplinary nature of the faculty was especially valuable for topics like engagement of interest holders and hospital-based HTA.

The training participants were selected based on their engagement in HTA and MD-related activities. This included professionals from the HTA Department of the State Expert Center of the MOH, nongovernmental HTA agencies, representatives of the MD industry and business associations, as well as experts from academic and research institutions. This inclusive approach ensured broad outreach and captured the diversity of interest holders within the HT ecosystem.

## Participants’ assessment and feedback

Assessment was a core component of the program. All participants completed a baseline knowledge assessment (a 21-item questionnaire designed by the lecturers) at the start of the course. At its conclusion, they took the same test to measure learning gains. Participants were evaluated according to their performance in the post-training survey. A threshold of 75 percent was established before the training, in accordance with SAFEMed standards for demonstrating sufficient competency in the training objectives. Participants scoring at or above this threshold were considered to have successfully completed the training program and received a certificate.

The post-training survey also gathered qualitative feedback on content, lecturers, logistics and overall experience to evaluate participant satisfaction and identify areas for improvement in future training programs.

## Program delivery and results

The training program was delivered over 4 months, from September to December 2024, enrolling 71 participants and 41 hours of virtual face-to-face lectures. The modular-based curriculum covered diverse topics such as economic evaluation, life cycle approaches, adaptation of HTA reports, engagement of interest holders and the use of real-world data. Innovative topics like HTA for digital HTs and emergency preparedness were also integrated to reflect global priorities (Table 1).

**Table 1.** Sessions of the online training program on health technology assessment of medical devices

Lecture title	Duration (h)
Opening session	0.5
Health technology assessment of medical devices	1
Assessing medical devices and pharmaceuticals: similarities and differences	1
The economic evaluation of medical devices	1.5
Case study – economic evaluation	1
Home task introduction – economic evaluation	0.5
Home task discussion and results – economic evaluation	1
Integration of HTA of medical devices into decision-making: hospital level	2
Horizon scanning and early HTA on medical devices	2
Organizational impact and health policy on medical devices	2
Case study – horizon scanning	1
Lifecycle approach for medical devices	1.5
Case study – life cycle approach	1
Home task introduction – life cycle approach	0.5
Home task discussion and results – life cycle approach	1
Disinvestment and HTA	1
Case study – disinvestment	0.5
Stakeholder involvement: patients	1.5
Stakeholder involvement: industry and healthcare management	1.5
Adaptation of the HTA of medical devices	1.5
Case study – HTA adaptation	1
Home task introduction – HTA adaptation	0.5
Home task discussion and results – HTA adaptation	1
Case study – complex technologies	1
HTA of medical devices in Central and Eastern Europe	1
HTA and digital health	1.5
A Model for Assessment of Telemedicine (MAST)	1
Practical session: HTA of digital health technology vs. standard of care	2
Medical devices and in vitro diagnostics: regulatory framework	2
Data sources for medical devices	1
*Invited lecturer – regulation of medical devices in Ukraine*	0.5
Case study – organizational impact	1
Quality of evidence for medical devices	1
HTA applied to emergency preparedness	2
Closing session	1
*Total number of hours*	*41*

Results of the post-training test indicated a significant increase in knowledge. The average correct response rate improved from 75 percent to 91 percent. The number of questions answered correctly by over 80 percent of participants rose from 7 to 20. For questions with over 90 percent accuracy, the number increased from 1 to 14. Of the 71 participants, 62 achieved a passing score of the established 75 percent threshold or higher and received a certificate of completion. Participants significantly improved their understanding of key HTA domains, particularly organizational and economic aspects, where methodologies differ from those used for pharmaceuticals. They also gained specialized competencies in evaluating digital health solutions. Feedback highlighted the program’s relevance, particularly in areas such as regulatory frameworks, economic evaluations and quality of evidence. Participant feedback was overwhelmingly positive, with high ratings for content relevance, instructional quality and practical applicability. Suggestions for improvement included more interactive workshops and follow-up sessions.

## Discussion

This paper presents the design and outcomes of a national training program specifically focused on strengthening competencies among Ukrainian professionals in the HTA of MD to complement the existing capacity on the HTA of pharmaceuticals. In this context, competencies are defined as a combination of knowledge, skills and attitudes that enable an individual to perform tasks and responsibilities effectively and to an established standard within a specific context. The course was explicitly designed to build such competencies in the field of HTA with a focus on MD.

This is acknowledged as a necessary add-on considering the lack of comparative evidence at launch, the rapid iterative changes and the nuanced evaluation of organizational impacts, which are all typical of MD.

The findings highlight the importance of structured, competency-based training programs in strengthening HTA capacity (12). As Ukraine continues its healthcare reforms, especially as it moves toward mandating HTA for MD, local expertise will be essential for ensuring cost-effective adoption of new HT, supporting evidence-based reimbursement decisions and aligning national policies with EU HTA frameworks.

A key limitation of this training program relates to its online delivery format. While hybrid and online training models have become commonplace and are documented as effective – particularly when supported by measures such as mandatory attendance requirements – this course was fully online due to the specific context of Ukraine amid the ongoing conflict. This format inevitably limited participant accountability for attending all sessions and fully engaging throughout the program. Additionally, the virtual setting constrained opportunities for spontaneous interaction between participants and facilitators, which are often more easily fostered in in-person environments.

Despite these challenges, online courses offer valuable flexibility and broad accessibility. To mitigate the drawbacks of remote learning, the course incorporated hands-on, interactive components that facilitated peer exchange and practical application of knowledge with the aim of maximizing learning outcomes (13). In particular, the use of real-world case studies proved valuable in helping participants apply theoretical concepts to practical scenarios. Participants were drawn from across Ukraine and represented a wide range of sectors, including industry, academia, international organizations, governmental institutions, consultancy firms and hospitals. This diversity enriched discussions and facilitated peer-to-peer learning, while also contributing to the development of a stronger HTA network within the country. However, such heterogeneity may have introduced variability in participants’ baseline knowledge, expectations and learning needs. While this enriched the learning environment, it may have also posed challenges in delivering uniformly effective training and in evaluating the impact of the course across different professional subgroups.

In addition, the use of real-world case studies served as a critical tool in bridging the gap between theoretical knowledge and practical competencies, enabling participants to apply core HTA principles in realistic scenarios. Future programs should incorporate mentorship and follow-up workshops to reinforce learning, as well as performance assessments 1 year post-training to determine the extent of knowledge retention and identify whether additional strategies are needed to sustain expertise in emerging HTs and the latest HTA methodologies. The successful implementation of this online training program demonstrates that targeted capacity-building efforts can significantly enhance MD HTA expertise in Ukraine and other similar contexts.

## Conclusions

This online training program successfully addressed a key gap in Ukraine’s HTA ecosystem by building competencies in MD evaluation. The initiative improved participant knowledge, promoted application of international best practices and created a foundation for future capacity-building efforts. As Ukraine advances its healthcare reforms and aligns with EU HTA practices, such training programs will be crucial for reinforcing its MD assessment framework. The positive outcomes of this initiative suggest that similar programs could be replicated in other transitioning health systems. For long-term impact, Ukraine should invest in continuous professional development, mentorship schemes and formal academic integration of HTA training. This program marks an important milestone toward achieving a robust, evidence-informed system for MD adoption and reimbursement in Ukraine.
